# Distinguishing Major Depression from Bipolar Disorders: DSM-5 Dimensional Model and Depression-related Psychotic Features

**DOI:** 10.1192/j.eurpsy.2026.11004

**Published:** 2026-07-31

**Authors:** R. Carvalho, J. Gama-Marques, J. Henriques-Calado

**Affiliations:** 1 Faculdade de Psicologia, Universidade de Lisboa, Lisboa, Portugal; 2 Consulta de Esquizofrenia Resistente, Hospital Júlio de Matos, Unidade Local de Saúde São José, Centro Clínico Académico de Lisboa, Lisboa, Portugal; 3 Clínica Universitária de Psiquiatria e Psicologia Médica, Faculdade de Medicina, Universidade de Lisboa, Centro Académico de Medicina de Lisboa, Lisboa, Portugal; 4 Centro de Investigação em Ciência Psicológica (CICPSI), Faculdade de Psicologia, Universidade de Lisboa, Lisboa, Portugal

## Abstract

**Introduction:**

Psychotic manifestations in depressive episodes are present in both Major Depressive Disorder (MDD) and Bipolar Disorder (BD). However, empirical research employing dimensional models has been largely restricted to psychotic characteristics when distinguishing between depressive dimensions in MDD and BD.

**Objectives:**

To explore how psychotic features, in interaction with dimensional personality traits and psychopathological symptoms, delineate MDD from BD.

**Methods:**

A cross-sectional study included 168 psychiatric patients (MDD=101, BD=67). Assessments: Personality Inventory for DSM-5 (PID-5), NEO Five-Factor Inventory (NEO-FFI), Brief Symptom Inventory (BSI). ANOVA and regression analyses were conducted.

**Results:**

MDD patients showed higher levels of anhedonia (*F*=10.71, *p*<.001), depressivity (*F*=7.89, *p*<.01), depression, somatization, anxiety and paranoid ideation (all *p*<.05) compared to the BD cohort and reported lower extraversion (*F*=5.15, *p*<.05). The mean scores for the PID-5 psychoticism facets (eccentricity, cognitive-perceptual dysregulation, and unusual beliefs) did not differ significantly. However, predictive models revealed stronger effects: in MDD, cognitive-perceptual dysregulation predicted depressive symptoms (β=.47, *p*<.001), while unusual beliefs negatively predicted depressivity (β=-.20, *p*=.01). In BD, cognitive-perceptual dysregulation was also significant (β=.50, *p*<.001), alongside interpersonal instability features.

**Conclusions:**

MDD is characterized by anhedonia, depressivity, low extraversion, and psychotic experiences such as paranoid ideation. These traits align MDD with the psychosis spectrum, with anhedonia as a potential vulnerability marker. Elevated paranoid ideation links depression and psychosis, suggesting an affective-paranoia continuum. The overlap of severe MDD and psychotic features indicates shared vulnerability pathways, while BD is less defined by symptoms. DSM-5 traits help refine depressive disorder boundaries. These findings suggest that cognitive-perceptual dysregulation represents a transdiagnostic vulnerability marker, while unusual beliefs and paranoid ideation may help distinguish depressive profiles between disorders.

**Disclosure of Interest:**

None Declared

